# Increased AMP‐activated protein kinase in skeletal muscles of Murphy Roth Large mice and its potential role in altered metabolism

**DOI:** 10.1002/phy2.252

**Published:** 2014-03-20

**Authors:** Tirsit K. Berhanu, Jenan Holley‐Cuthrell, Nathan W. Roberts, Aaron J. Mull, Ahlke Heydemann

**Affiliations:** ^1^ Department of Physiology and Biophysics The University of Illinois at Chicago Chicago Illinois; ^2^ Center for Cardiovascular Research The University of Illinois at Chicago Chicago Illinois

**Keywords:** AMP‐dependent protein kinase, metabolic flux analysis, mitochondria, Murphy Roth Large, skeletal muscle

## Abstract

Wild‐type Murphy Roth Large (MRL) mice have long been investigated for their superior healing ability when subjected to various wound and disease models. Despite this long history, the mechanisms causing their extraordinary healing ability remain undefined. As we have recently demonstrated that MRL mice with muscular dystrophy are resistant to the associated fibrosis and the Heber‐Katz group has demonstrated MRL mitochondrial mutations, we decided to investigate the skeletal muscle metabolic characteristics of the MRL mouse strain compared to the commonly utilized C57BL/6J control mouse strain. We now have evidence demonstrating an altered metabolism in the MRL quadriceps, triceps brachii, and diaphragm of 8‐week‐old animals compared to tissues from control animals. The MRL skeletal muscles have increased activated phosphorylated AMP‐activated protein kinase (pAMPK). The increased pAMPK signaling coincides with increased skeletal muscle mitochondrial content. These metabolic changes may compensate for insufficient oxidative phosphorylation which is demonstrated by altered quantities of proteins involved in oxidative phosphorylation and *ex vivo* metabolic investigations. We also demonstrate that the MRL muscle cells have increased metabolic physiologic reserve. These data further the investigations into this important and unique mouse strain. Why the MRL mice have increased pAMPK and how increased pAMPK and the resultant metabolic alterations affect the healing ability in the MRL mouse strain is discussed. Understanding the molecular mechanisms surrounding the super healing characteristics of these mice will lead to relevant clinical intervention points. In conclusion, we present novel data of increased mitochondrial content, pAMPK, and glycolytic indicators in MRL skeletal muscles.

## Introduction

The unique Murphy Roth Large (MRL)/MpJ scar‐less wound healing abilities were first noted when ear punches used for identification closed and fully, scarlessly, healed within 4 weeks (Clark et al. 1998). Many, but not all, subsequent MRL wound models also display this unique healing ability (reviewed in (Heydemann 2012)). The MRL/MpJ‐*Fas* ^Lpr^/J mouse strain has a homozygous null mutation in the *fas* gene which causes decreased apoptosis in lymphocytes (Juvet et al. 2013). The mouse strain used in the current work is the MRL/MpJ strain (MRL), which has an intact *fas* antigen gene (Adachi et al. 1993). Both the MRL/MpJ and MRL/MpJ‐*Fas* ^Lpr^/J mouse strains have increased ear punch hole healing and both are prone to specific autoimmune diseases, although the MRL/MpJ mice succumb to autoimmune disease later in life (Theofilopoulos and Dixon 1985); well passed the 8‐week‐old mice used in this study. Therefore, we can surmise that the *fas* mutation is not necessary for the autoimmune phenotype, and additional genes in the MRL mouse strains contribute to the autoimmune phenotype.

The molecular and cellular mechanisms behind the MRL healing ability have not been fully elucidated. There are multiple well‐supported hypotheses (reviewed in (Heydemann 2012)). Among the possible, and in no way mutually exclusive, ideas are; enhanced stem cell abilities (Baker et al. 2006; Naviaux et al. 2009), altered cell cycle regulation (Arthur et al. 2010; Bedelbaeva et al. 2010), immune differences (Donnelly et al. 1990; Alleva et al. 1997; Kench et al. 1999; Ueno et al. 2005), alterations in the extra‐cellular matrix (Heber‐Katz et al. 2004) and, metabolic differences (Naviaux et al. 2009).

We hypothesize, and have preliminary evidence which supports metabolic differences as an explanation for a significant portion of the MRL healing phenotype. MRL mitochondria have been shown to contain two significant heteroplasmies (multiple mitochondrial genomes present in a single cell, (Sachadyn et al. 2008)). Mitochondria contain multiple plasmid‐like genomes which replicate and segregate randomly during fission. In the MRL strain some of these mitochondrial genomes contain mutations. The percentage of mutant mitochondrial genomes is different between animals and a threshold of mutant genomes is required for phenotypic penetrance confounding research into this discipline. The two MRL mitochondrial heteroplasmies contain mutant nucleotide sequences in mitochondrial tRNAs and are expected to cause variable and multiple translational errors. In a previous example, Ghezzi et al. identified that alteration in a mitochondrial tRNA modifying enzyme causes hypertrophic cardiomyopathy and lactic acidosis (Ghezzi et al. 2012). Similarly, mitochondrial tRNA mutations can cause hypertension in humans (Qiu et al. 2012). Although these example mutations are in different mitochondrial tRNA genes we can deduce that the MRL mitochondrial tRNA mutations also cause translational errors. These mitochondrial translational errors will lead to the metabolic alterations identified in MRL fibroblasts, MRL blastema‐derived fibroblasts, and MRL heart tissue (Naviaux et al. 2009) and to the currently identified metabolic alterations in the MRL skeletal muscles.

A central regulator of many aspects of cellular metabolism is AMP‐dependent protein kinase (AMPK, reviewed in (Hardie 2011)). AMPK is activated by kinases when cells become energetically stressed; the activation is rendered allosterically possible by an increase in the AMP/ATP ratio, which also renders phosphatases inactive (Davies et al. 1995; Ponticos et al. 1998). In general, AMPK activation induces increases in catabolism and decreases in anabolism, both intended to restore energy homeostasis. Although alternative AMPK activators exist, our data indicate that insufficient ATP levels due to inadequate mitochondrial function are the activators in the MRL skeletal muscle tissue.

In light of the cardiac metabolic differences (including increased glycolysis and reduced fatty acid oxidation) identified by Naviaux et al. (2009) and to further investigate the MRL regeneration abilities, we now show that multiple skeletal muscle types (quadriceps, triceps brachii, and diaphragm) of wild‐type MRL mice have increased activated, phosphorylated, AMPK (pAMPK), and altered levels of mRNA and proteins involved in mitochondrial biogenesis and oxidative phosphorylation when compared to the commonly utilized C57BL/6J (B6) control mice. From previously published work and these new, novel data we present a model of MRL metabolic differences followed by a discussion of how these metabolic differences increase the regenerative abilities of the MRL mice.

## Materials and Methods

### Mice

Murphy Roth Large (MRL)/MpJ (MRL, 000486) and C57BL/6J (B6, 000664) male mice were purchased from the Jackson Laboratories (Bar Harbor, ME). The mice were housed 5 to a cage in the same room of a barrier facility, with unrestrained access to standard mouse chow and neutral water, and 12‐h light/dark cycles. All protocols were conducted adhering to the rules of the National Institutes of Health Guide for the Care and Use of Laboratory Animals and by the University of Illinois at Chicago Institutional Animal Care and Use Committee.

### Immunoblots

Tissues intended for immunoblots were harvested and snap frozen in liquid nitrogen. Proteins were prepared in 500 *μ*L of lysis buffer (20 mmol/L HEPES [pH 7.4], 50 mmol/L *β*‐glycerol phosphate, 2 mmol/L EGTA, 1 mmol/L DTT, 10 mmol/L sodium fluoride, 1 mmol/L sodium orthovanadate, 1% TritonX‐100, 10% glycerol and protease inhibitors [ThermoScientific, 1862209, Waltham, MA]) with homogenization TissueRuptor (Qiagen) on ice, at full speed for 1 min. After centrifugation at 4500 *g* for 10 min the protein concentrations of the supernatants were quantified with the Bradford assay (ThermoScientific). Acrylamide gels (BioRad, Hercules, California) were used to separate 30–60 *μ*g of protein, and then transferred to PVDF membrane (iBlot, Invitrogen, Carlsbad, CA). Immunodetection was performed at room temperature; the membrane was blocked for 20 min in 5% nonfat dry milk in t‐TBS (Tris‐Buffered Saline with 0.1% Tween 20), incubated for 2 h in primary antibody [voltage‐dependent anion channel 1 (VDAC1; AbCam, Cambridge, MA), pAMPK and AMPK (both antibodies recognize both *α*1 and *α*2 subunits and both are from Cell Signaling Technology #2535 and #2793, respectively), GLUT4 (Millipore, Billerica, MA), and OXPHOS (AbCam ab110413) diluted 1:1000 in 5% nonfat dry milk in t‐TBS, washed three times 15 min in t‐TBS, incubated for 1 h in appropriate secondary horseradish linked antibody (Jackson Immunologicals, Carlsbad, CA)] diluted 1:2500 in 5% nonfat dry milk in t‐TBS, washed three times 15 min in t‐TBS, developed with ECL (GE Healthcare, Little Chalfont, Buckinghamshire, UK), and visualized with a ChemiDoc (Bio Rad, Hercules, CA). Gels were stripped at 55°C for 30 min in 2% SDS, 100 mmol/L mercaptoethanol, 62.5 mmol/L Tris‐HCL pH 6.8, and reprobed with GAPDH (1:3000; Santa Cruz Biotechnology, Santa Cruz, CA) for a loading control. Immunoblot bands were quantified with ImageJ, and the results are reported as a ratio of the GAPDH lane. Multiple exposures were used to quantify gels with a wide range of protein levels. In all cases the B6 average from the gels was set to represent 100% and the other bands were compared as ratios to this normalization. The number of biological replicates quantified for each strain are indicated in the figure legends.

### Quantitative PCR (qPCR)

Quantitative mitochondrial DNA and nuclear DNA contents were analyzed as described (Moreno‐Loshuertos et al. 2006). Small portions (~20 mg) of tissue were prepared with the Puregene kit (Qiagen, Hilden, Germany) as per the product protocol except that the final resuspension occurred in 30 *μ*L. The mitochondrial encoded Subunit II of Cytochrome C Oxidase was amplified with forward primer 5′‐CTACAAGACGCCACAT and reverse primer 5′‐GAGAGGGGAGAGCAAT. PCR product intensity was compared to the single nuclear gene encoding Succinate Dehydrogenase Subunit A intensity, amplified with forward primer 5′‐TACTACAGCCCCAAGTCT and reverse primer 5′‐TGGACCCATCTTCTATGC. The PCR conditions were as follows: 95°C 10 min; then [95°C, 15 sec and 60°C, 1 min] times 40 cycles in a Rotor‐Gene Q (Qiagen). Melt curves were utilized to verify a single product produced in each reaction, and dilution curves were generated to identify the efficiency of each primer pair. The ratio of the two products was determined using ^Δ^C^t^ comparisons for each animal. There is no reverse transcription step: we are comparing mitochondrial to nuclear DNA contents directly. We set the B6 animal ratios to 100% and report the MRL ratios as a percentage of this.

### Citrate synthase activity

The assay was performed according to manufacturer's instructions (Sigma, St. Louis, MO). Six quadriceps samples were processed from each strain. Results are expressed as citrate synthase umol/min/mg mitochondrial extract.

### ATP assay

The colorimetric assay was performed according to manufacturer's instructions (BioVision, Milpitas, CA). Four quadriceps samples were processed from each strain.

### QuantiGene

Small portions (10–30 mg) of tissues were flash frozen in liquid nitrogen. The samples were prepared according to manufacturer's instructions (QuantiGene Affymetrix, Santa Clara, CA) and delivered to the DNA Services Facility section of The University of Illinois at Chicago Research Resources Core Facility. The samples were run in duplicate on a custom designed assay plate. At least eight biological replicates were performed. The control transcript, transferrin receptor protein 1 (TfR1) was chosen as the control gene from a set of six potential control genes because TfR1 had the lowest standard deviation within the two animal groups but had sufficient expression (at least 50% of the control RNA sample). Therefore, all of the mRNA species were normalized to the TfR1 (transferrin receptor protein 1) level from that same sample. The expression levels of all genes, including the controls are investigated in a single reaction. These assays were conducted upon quadriceps (*n* = 8) and diaphragm (*n* = 10) tissues from the MRL and B6 mouse strains. To graph the results the data were again normalized to the average B6 expression level of the specific gene, so that results can be illustrated on one graph as a percentage, and the trends between genes can be easily viewed.

### Primary cell culture

Primary neonatal mouse myoblasts were isolated with a modification of previously described methods (Rando and Blau 1994) as follows. Briefly, limb muscles were removed from P0 to P2 pups, deskinned, and minced. The slurry was pelleted and resuspended in 1 mg/mL collagenase/dispase (Roche Diagnostics Corporation, Indianapolis, IN) in Ham's F10 at 37°C for 45 min, triturating every 15 min. Digests were passed through a 100‐*μ*m cell strainer, pelleted, and resuspended in primary myoblast medium (Ham's F10, 20% FBS, 1% PSA (all media Gibco, Grand Island, NY) and 2.5 ng/mL recombinant human bFGF (Promega, Madison, WI)). Myoblasts were preplated for 10 min on uncoated dishes and then nonadherent cells (enriched myoblasts) were plated on gelatin‐coated dishes (2% gelatin type B [Sigma]) at 37°C, 5% CO_2_. Proliferation abilities were assessed by plating an equal number of cells into wells of a six‐well gelatin‐coated plate. The cells were given fresh media every 24 h and harvested with trypsin at 72 h after plating. The cells were carefully counted and mitosis/24‐h period was calculated for each culture. Cultures from six different animals of each strain were assessed. In addition, routine desmin antibody (1:100 dilution, primary antibody from Santa Cruz Biotechnology, Alexa Fluor goat anti‐mouse 488 secondary antibody, Life Technologies) staining was performed on cultures used for the metabolism analysis.

### Seahorse metabolic flux analysis

To gain further insight into the metabolic characteristics of the MRL skeletal muscles, primary isolated myoblast progenitor cells were analyzed in the Seahorse apparatus (Seahorse Biosciences, North Billerica, MA). Preliminary data indicated that 30,000 primary myoblast cells per well of the Seahorse plate yielded the most reproducible results for both primary cell lines. For oxygen consumption rate (OCR)/extracellular acidification rate (ECAR) experiments, cells frozen once and thawed were plated on coated or uncoated XF‐24 plates (Seahorse Bioscience, North Billerica, MA) at 30–40,000 cells overnight, fed 675 *μ*L Seahorse unbuffered assay media (modified DMEM, no bicarbonate or pyruvate, 4.5 g/L glucose) and the assay was performed as described in manufacturer's instructions with concentrations listed. The cells were treated with 1 *μ*mol/L voligomycin, 500 nmol/L carbonyl cyanide p‐trifluoromethoxyphenylhydrazone (FCCP), and 1 *μ*mol/L antimycin and both OCR and ECAR measured in a Seahorse Extracellular Flux Analyzer. Then three experiments were conducted using three separate primary cultures from each mouse strain. Each experiment contained at least six wells of myoblasts from each strain. Values for metabolic responses were calculated by areas under the curve.

### Statistics

As we are comparing two populations, MRL versus B6, student's *t*‐tests were performed, and significant differences were considered when *P* < 0.05 (Excel Software; Microsoft, Seattle, WA).

## Results

### MRL skeletal muscle tissues have increased mitochondrial content

**Figure 1:**
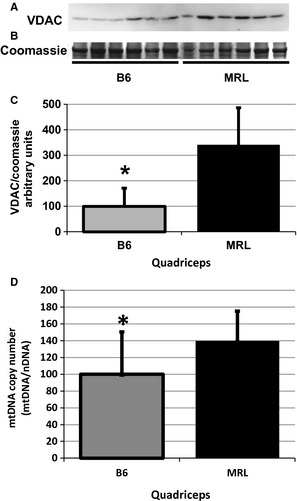
Increased mitochondrial markers in Murphy Roth Large (MRL) quadriceps muscle tissue. (A) VDAC1 (porin) specific antibody demonstrates significantly increased mitochondrial content in MRL quadriceps. (B) Coomassie stain of a duplicate gel demonstrating equal loading. (C) VDAC was quantified from six biological replicates and normalized to the coomassie stain. (D) Quantitative PCR demonstrates a significant increase in the mitochondrial to nuclear DNA ratio in the MRL quadriceps tissues. Twelve quadriceps from each strain were analyzed and compared, averages with standard deviations are shown. *represents *P* < 0.05 versus the MRL mice.

Previously reported qPCR results demonstrated an approximately twofold increase of mitochondrial to nuclear DNA ratios of MRL liver and cardiac tissues compared to B6 tissues (Naviaux et al. 2009). Similarly, electron microscopy demonstrated increased mitochondrial content in the MRL muscle tissues versus the 129 control strain (Heydemann 2012). As the mitochondrial content in skeletal muscle is dependent upon individual fiber type, the previous electron microscopy results are now supported by immunoblots of the VDAC1, also known as the mitochondrial porin protein levels. MRL quadriceps muscles contained significantly more VDAC protein then the controls (representative blot shown in Fig. 1A and quantified in Fig. 1B [*n* ≥ 10]). The diaphragms and triceps brachii of the MRL mice also had increased VDAC protein levels (Data not shown). The differences were all statistically significant between the MRL and B6 controls: quadriceps (MRL 340 ± 147 standard deviations, B6 100 ± 72, *P* = 0.0048), diaphragms (MRL 189 ± 69, B6 100 ± 52, *n* = 6, *P* = 0.0372), and triceps brachii (MRL 183 ± 126, B6 100 ± 83, *n* = 12 *P* = 0.037).

**Figure 2:**
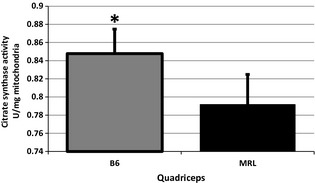
Citrate synthase activity is decreased in Murphy Roth Large (MRL) muscles. Six quadriceps from each strain were analyzed and compared, averages with standard deviations are shown. *represents *P* < 0.05 versus the MRL mice.

To further confirm the increased quantity of mitochondria in MRL muscles, we employed qPCR comparing mitochondrial DNA to nuclear DNA ratios. The MRL quadriceps muscle tissue demonstrated increased ^Δ^C^t^ ratios of mitochondrial to nuclear DNA compared to the control strain (Fig. 2A, MRL 139 ± 36, B6 100 ± 50, *n* = 12, *P* = 0.018). The same analysis was performed upon DNA extracted from diaphragm and again the MRL mice had significantly increased ^Δ^C^t^ ratios (Data not shown, MRL 195 ± 42, B6 100 ± 27, *n* = 12, *P* = 0.0004 MRL vs. the B6).

We also utilized the citrate synthase activity assay, a commonly utilized method for mitochondrial quantification. The B6 quadriceps had significantly higher levels of citrate synthase activity than the MRL quadriceps tissue (Fig. 2B, (MRL 0.848 ± 0.027, B6 0.792 ± 0.033, *P* = 0.012, *n* = 6). This apparent discrepancy will be clarified in the discussion.

### Increased transcripts of genes involved in mitochondrial biogenesis

**Figure 3:**
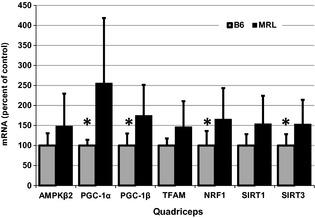
Transcripts for mitochondrial biogenesis are increased in Murphy Roth Large (MRL) quadriceps tissue. QuantiGene analysis indicates increases of key regulatory factors of mitochondrial biogenesis in MRL muscles. Nine muscles from each strain were compared and normalized to an internal control. The values were then normalized to the B6 values; averages with standard deviations are shown. AMPK *β*2, AMP‐activated protein kinase beta 2 subunit; PGC‐1*α* and 1*β*, peroxisome proliferator‐activated receptor gamma coactivators 1‐alpha; and 1‐beta; transcription factor A, mitochondrial transcription factor A; NRF1, nuclear respiratory factor 1; SIRT1 and SIRT3, Sirtuin1 and 3. *represents *P* < 0.05 versus the MRL mice.

Mitochondrial biogenesis requires the coordinated interactions of multiple transcription factors and their downstream targets. To investigate if these mitochondrial biogenesis regulating transcripts are significantly altered in the skeletal muscles of MRL mice QuantiGene assays were performed. Compared to B6 controls, quadriceps muscles of MRL mice had increased mRNA levels for all of the seven mitochondrial biogenesis factors interrogated (Fig. 3). Despite the large standard deviations provided by the MRL data, the differences were statistically significant for peroxisome proliferator‐activated receptor gamma coactivator 1‐alpha (PGC‐1*α*), PGC‐1*β*, nuclear respiratory factor (NRF1), and sirtuin3 (SIRT3); (*P* = 0.017, 0.021, 0.047, 0.039, respectively). In the QuantiGene analyzed MRL diaphragm tissues, only the mRNA for AMPK*β* was significantly increased (*P* = 0.015), and the MRL diaphragms actually had decreased trends for the PGC‐1*α*, PGC‐1*β*, transcription factor A (TFAM), SIRT1 and SIRT3 expression levels compared to B6 controls (Data not shown).

### MRL skeletal muscles have increased activated AMPK protein levels

**Figure 4:**
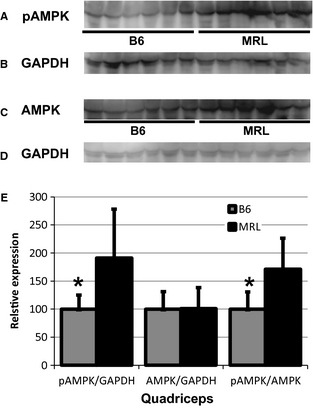
Increased phosphorylated AMP‐activated protein kinase (pAMPK) and AMPK in Murphy Roth Large (MRL) quadriceps muscle. (A) A pAMPK‐specific antibody demonstrates increases in MRL quadriceps. (B) GAPDH loading control of same gel. (C) AMPK‐specific antibody analyzing quadriceps tissue. (D) GAPDH loading control of same gel. (E) Activated pAMPK over AMPK ratio, arbitrary units, normalized to B6 control levels, averages with standard deviations are depicted. *n* = 9, *represents *P* < 0.05 versus MRL tissue.

We next sought to investigate a molecular mechanism leading to increased mitochondrial content. Because of their mitochondrial heteroplasmies (Naviaux et al. 2009), we hypothesize that the MRL mice are being challenged with a chronic decrease of cellular energy, which would cause the cells to compensate by increasing mitochondrial quantity. Initially, the cell identifies decreased energy by an increase in the AMP to ATP ratio, which allows activation of AMPK through sustained phosphorylation at Thr‐172. Phosphorylated AMPK (pAMPK) has multiple downstream effects including increased mitochondrial content (Zong et al. 2002). We now find that the MRL quadriceps tissues have significantly increased pAMPK (representative quadriceps immunoblot Fig. 4A, GAPDH loading control 4B, and quantified in 4C, *P* = 0.0315), and no change in AMPK levels (representative quadriceps immunoblot Fig. 4D and quantified in 4E, *P* = 0.935). Furthermore, the ratios of pAMPK over total AMPK are significantly increased in the MRL quadriceps tissue compared to the B6 muscles (Fig. 4F, *P* = 0.0175).

The increases of pAMPK and AMPK protein levels in MRL diaphragms and triceps brachii are also noteworthy. The MRL diaphragms demonstrated significantly increased pAMPK and AMPK levels (Data not shown, pAMPK *P* = 0.005, AMPK *P* = 0.016). The ratio of activated to unphosphorylated AMPK is slightly increased in the MRL diaphragm tissue (*P* = 0.482). The MRL triceps brachii have significantly increased AMPK and trending to an increased pAMPK (Data not shown; pAMPK *P* = 0.089, AMPK *P* = 0.034). In the triceps brachii tissue, the pAMPK/AMPK ratio is significantly increased in the MRL mouse tissue compared to the B6 control (*P* = 0.028).

We also assessed ATP levels in quadriceps tissue from the two mouse strains. No significant difference was detected between the two mouse strains (MRL 0.013 ± 0.0078 mmol/L ATP/mg protein, B6 0.009 ± 0.0029, *n* = 4, *P* = 0.417).

### MRL skeletal muscles have increased metabolic transcripts and proteins

**Figure 5:**
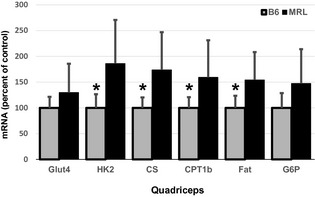
Increased metabolic transcripts in Murphy Roth Large (MRL) quadriceps. Nine muscles from each strain were compared via QuantiGene and normalized to an internal control. The values were then normalized to the B6 values; averages with standard deviations are shown. GLUT4, glucose transporter type 4; HK2, hexokinase 2; CS, citrate synthase; CPT1b, carnitine palmitoyltransferase 1; FAT, FAT/CD36; G6P, glucose 6‐phosphate. *represents *P* < 0.05 versus the MRL mice.

Increases in pAMPK initiate multiple downstream signaling cascades. In particular, we are interested in metabolic differences in the MRL mice and therefore have analyzed the levels of mRNAs and proteins involved in cellular glycolysis and mitochondrial oxidative phosphorylation. By QuantiGene analysis we identified a significant increase in four metabolic enzyme mRNA transcripts in MRL quadriceps in (Fig. 5). These significant increases were found for hexokinase 2 (HK2, *P* = 0.016), citrate synthase (CS, *P* = 0.016), carnitine palmitate 1b (CPT1b, *P* = 0.042), and fatty acid translocase (Fat/CD36, *P* = 0.021). When diaphragm tissues were compared (Data not shown) we found significant increases of glucose transporter 4 (GLUT4, *P* = 0.021) and CS (*P* = 0.0421) in MRL tissues.

By immunoblot of membrane fractions we also identified an increase in the protein product of GLUT4 in the membrane fraction of MRL quadriceps muscle (data not shown, values normalized to caveolin 3, MRL 155 ± 23, B6 100 ± 13, *n* = 6, *P* = 0.0057). This demonstrates an increase in a well‐known pAMPK target.

### Alterations in protein content of electron transport chain complex members

An oxidative phosphorylation (OXPHOS) antibody cocktail was used to quantitatively assess the changes in ETC proteins in the quadriceps muscle samples. The MRL mice were expected to have increased amounts of all complexes as they have more mitochondria. Interestingly, only the nuclear encoded Complex V protein, ATP5A was increased in MRL quadriceps (data not shown, normalized to GAPDH, MRL 189 ± 69, B6 100 ± 52, *n* = 4, *P* = 0.0372). The diaphragm OXPHOS immunoblots revealed no significant differences between the MRL and B6 mouse strains (data not shown).

### Increased glycolysis and oxidative phosphorylation identified in MRL primary muscle cells

**Figure 6:**
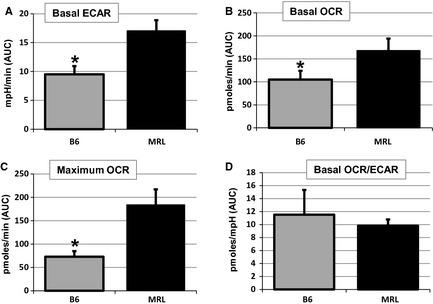
Metabolic differences in isolated primary muscle progenitor cells. Measurements of oxygen consumption rate (OCR) and extracellular acidification rate (ECAR) with a Seahorse XF24 Flux Analyzer reveal metabolic differences between Murphy Roth Large (MRL) and B6 cells. (A) Basal ECAR indicates increased glycolysis in MRL muscle progenitor cells. (B) Basal OCR is also significantly higher in MRL cells. (C) MRL cells have an increased physiologic reserve as demonstrated by the significantly increased maximal OCR after carbonyl cyanide p‐trifluoromethoxyphenylhydrazone (FCCP) mediated uncoupling of the membrane potential. (D) Slightly decreased basal OCR/ECAR indicates MRL muscles do indeed favor glycolysis. * represents *P* < 0.05 versus the MRL cells.

Supporting the mRNA expression data and previously reported data for other MRL tissues (Naviaux et al. 2009), we now show that MRL primary muscle progenitor cells perform more glycolysis than B6 progenitor cells. Using the Seahorse Metabolic Flux Analyzer we identified that the MRL cells have significantly increased acidification of their media indicating increased lactic acid levels, a definitive end product of anaerobic glycolysis (Fig. 6A). The metabolic data also indicate that the MRL skeletal muscles perform more basal oxidative phosphorylation demonstrated by increased oxygen consumption (Fig. 6B). MRL muscle progenitor cells also demonstrate increased maximal oxygen consumption after FCCP treatment, which permeabilizes the inner mitochondrial membrane to protons, thus it represents the maximum oxygen that the mitochondria can utilize as final electron acceptor (Fig. 6C). The MRL muscle cells also have a not significantly decreased ratio of oxygen consumption to acidification rate (Fig. 6D). This further indicates that the MRL muscle cells favor glycolysis over oxidative phosphorylation.

To substantiate these metabolic quantifications, we confirmed that the primary muscle cultures were similar. There was no difference between the MRL and B6 cultures in regards to proliferation rates. Furthermore, all cultures were similarly positive (approximately 90% positive) for desmin staining indicating similar muscle precursor status.

## Discussion

### Integrative MRL metabolism model

**Figure 7:**
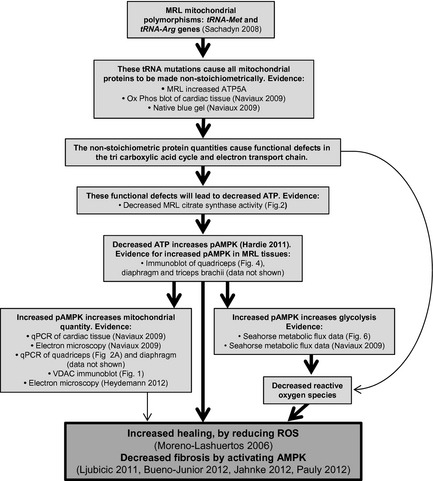
Schematic model of Murphy Roth Large (MRL) unique metabolic characteristics. Combining data from previously published results and current findings we propose this schematic model to aid in future investigations.

We have generated a working model of metabolism differences in the MRL mice, integrating previously published and current data (Fig. 7). The MRL mice are known to have two nonsynonomous mitochondrial heteroplasmies (cells containing both normal and mutant mitochondrial DNA copies, (Sachadyn et al. 2008)). Because the heteroplasmies occur in mitochondrial tRNAs, we expect the mitochondrial encoded proteins of oxidative phosphorylation will be made at reduced levels (Irwin et al. 2012; Szczepanowska et al. 2012) and, therefore, in nonstochiometric amounts compared to the nuclear encoded mitochondrial destined proteins. This leads to decreased efficiency of oxidative phosphorylation, resulting in decreased ATP levels. The cell compensates with increased activated AMPK, and the resulting increased mitochondrial quantity and noted altered metabolism. How the change in activated AMPK‐mediated metabolism may increase the regenerative capacity of these mice is yet to be determined. An increase in pAMPK through pharmaceuticals has been demonstrated a number of times to benefit animals with chronic muscular dystrophy associated necrosis and fibrosis (Ljubicic et al. 2011; Jahnke et al. 2012; Pauly et al. 2012). The working model schematic indicates some important intervention points that can be manipulated in future experiments to identify if the metabolic differences are causative for increasing regeneration.

One of the downstream results of this altered metabolism is the observed decreased reactive oxide species (ROS, (Naviaux et al. 2009)). Usually mitochondrial dysfunction elicits increases in ROS (Gilliam et al. 2013). Again, the MRL mice behave unexpectedly, and again the mechanism(s) of MRL decreased ROS remain unknown. High ROS levels are well known to inhibit various cellular processes and are pathogenically associated with many diseases. However, low ROS levels are often utilized for cellular signaling (reviewed in (Droge 2002)). Therefore, the reduction of ROS may be beneficial to the MRL tissues, and may be a contributor to the super healing phenotype in the MRL mice.

AMP‐dependent protein kinase activation is known to benefit a muscular dystrophy mouse model (Pauly et al. 2012). These changes are similar to the increases of pAMPK we are currently uncovering in the MRL mouse strain. We have previously shown that the MRL mouse strain is resistant to muscular dystrophy (Heydemann et al. 2012). Combining these data, it is a logical hypothesis to consider the increased pAMPK as central to the super healing of the MRL strain.

### Increased mitochondrial content in MRL skeletal muscle

By three methods, we have demonstrated increased mitochondrial content in the skeletal muscles of the MRL mice. To corroborate the previously published electron microscopy images (Heydemann 2012), immunoblots with VDAC were performed upon quadriceps, triceps brachii, and diaphragm protein extracts. VDAC is located on the outer mitochondrial membrane. Although variably expressed between tissue types (Garcia‐Cazarin et al. 2011), it appears to be uniformly expressed within skeletal muscles (Taub et al. 2012), and is therefore a valid surrogate marker for mitochondrial density (Jahnke et al. 2012).

As a further indicator of mitochondrial content, qPCR was performed upon triceps brachii and diaphragm muscles. We identified significant (1.5 to twofold) differences in mitochondrial DNA (mtDNA) quantities between MRL and B6 tissues. Naviaux et al. demonstrated twofold and threefold mtDNA increases in liver and heart tissue, respectively (Naviaux et al. 2009). The differences between the Heber‐Katz and Heydemann laboratories may arise from gender differences (previously assayed females vs. our males), or age differences (10‐week vs. our 8‐week‐old mice), a difference in tissue type, or, most likely, a combination of the listed factors with the last being preeminent.

As a final indicator of increased mitochondria in the MRL muscles a citrate synthase activity assay was conducted upon quadriceps muscle. This activity assay is commonly used to quantify intact mitochondria. The MRL quadriceps muscles have a reduction in the activity of this enzyme despite having increased mitochondria by three other quantitative measures. We believe that this data are not inconsistent but rather indicates the hypothesized inefficiency of the MRL mitochondria. If, as hypothesized, the MRL citric acid cycle is not performing correctly a bottle neck would ensue and the activity of the usually rate‐limiting citrate synthase enzyme would decrease. We hypothesize that the nonstoichiometric amounts of the mitochondrial encoded proteins of the cycle would cause the cycle to be inefficient. The actual steps which are under performing will be investigated in the future.

Since we demonstrate increased mitochondria in the MRL muscle we expect an increase in mRNA species that are responsible for mitochondrial biogenesis. In general, this is what is revealed by QuantiGene analysis. Peroxisome proliferator‐activated receptor gamma coactivator 1‐alpha (PGC‐1*α*) is a well‐known control point for increasing mitochondrial content (Godin et al. 2012). The MRL skeletal muscles have significantly increased amounts of mRNA for this protein. For full activation PGC‐1*α* phosphorylation by AMPK and deacetylation by SIRT are required (Lagouge et al. 2006). By immunoblot we demonstrate an increase of pAMPK and by QuantiGene we demonstrate an increase of SIRT1 and SIRT3. SIRT3 also has ROS reducing abilities (Kong et al. 2010), low ROS is yet another characteristic found in the MRL mice (Naviaux et al. 2009). A slight increase of TFAM was also noted. This transcription factor is central to the expression of multiple mitochondrial genes including those required for replication (Kanki et al. 2004). Likewise, nuclear respiratory factor (NRF1) is an essential mitochondrial transcription factor (Virbasius and Scarpulla 1994) which is significantly increased in the MRL skeletal muscles. We have yet to investigate mitophagy in the MRL skeletal muscle. Decreased mitophagy would also contribute to the increased mitochondrial content in these muscles.

Of note, the QuantiGene differences are slight because we are investigating a mouse strain not a genetically manipulated mouse. The MRL mice are generally healthy, so large perturbations are unexpected. But these slight differences in mitochondria and metabolism could impact the mechanism of healing and push the MRL mice to their super‐healing phenotype without establishing unwanted side effects.

### AMPK is chronically activated in the MRL skeletal muscles

Activation of AMPK occurs in cells with reduced energy, often identified as an increase in the AMP/ATP ratio (Hardie 2003). Cellular energy can be reduced by ischemia (Kudo et al. 1995), exercise (Winder and Hardie 1996), calorie restriction (Salt et al. 1998), mitochondrial dysfunction (Trifunovic et al. 2005; Hiona et al. 2010), or through pharmaceuticals, such as metformin (Foretz et al. 2010) or AICAR (Corton et al. 1995). Molecularly AMPK can be phosphorylated and thus activated by LKB‐1; the activation is maintained by allosteric hindrances to phosphatases provided by a high AMP/ATP ratio. As the large differences in AMPK activation occur between sedentary MRL and B6 mice, we reason that basal metabolic stress is the primary and chronic AMPK activator. The MRL cells attempt to compensate for decreased ATP by increasing their mitochondrial content; this appears to be somewhat successful as ATP levels are not different from the B6 controls. However, some stress signals persist, such as phosphorylated AMPK. A tight feed‐back loop is envisioned where the slightest decrease in ATP activates the AMPK to increase mitochondria and ATP output. This chronic condition is clearly different than acute pAMPK stimulation. Although we failed to detect decreased ATP in the MRL skeletal muscle tissues by the colorimetric assay; this assay may not be sensitive enough as we expect the actual difference to be slight. More sensitive HPLC detection assays are planned.

### Altered MRL metabolism

The current work corroborates and extends previous work of altered metabolism in MRL fibroblasts and liver tissue (Naviaux et al. 2009). Now, by identifying increased pAMPK in the skeletal muscle tissue of the MRL animals, we have added an important mechanistic point in the MRL scheme of metabolic differences and phenotype (Fig. 7). The increased pAMPK can explain many metabolic alterations identified by Navieux et al. in fibroblasts; increased glycolysis, increased mitochondrial number, altered OxPhos protein levels, and lower membrane potential (Naviaux et al. 2009). We now corroborate and extend the results to skeletal muscles.

We also analyzed mRNA expression levels of multiple enzymes involved in metabolism. The MRL quadriceps muscles had significantly increased levels of four of the six enzymes analyzed. Hexokinase 2 is the predominant hexokinase isoform in skeletal muscle, phosphorylates glucose for the glycolytic and oxidative phosphorylation pathways and is upregulated by insulin (Irimia et al. 2012). Citrate synthase is also upregulated in MRL muscles; it is the first and rate‐limiting step of the citric acid cycle. The fact that transcripts of carnitine palmitoyltransferase 1b (CPT1b) and CD36 fatty acid translocase (CD36/FAT) are also increased in MRL muscle tissue suggests that free fatty acid metabolism is also increased. CPT1b is found on the outer mitochondrial membrane and transports long‐chain fatty acids into the mitochondria. CD36/FAT is involved in long‐chain fatty acid uptake by the cell (Lombardi et al. 2012). These data provide new insights into MRL metabolism; the citric acid cycle and fatty acid metabolism may also be increased in addition to the already known increase of glycolysis.

Metabolic flux analysis performed upon the primary skeletal muscle cells isolated from the MRL and B6 mice corroborates the increased glycolysis previously demonstrated in fibroblast cells (Naviaux et al. 2009). Glycolysis is increased as is demonstrated by the increase in ECAR, and oxidative phosphorylation may also be increased as is demonstrated by the increase in the oxygen consumption rate (OCR). Furthermore, the MRL muscle cells appear to have increased oxidative reserve capacity as is demonstrated by the maximum OCR after the addition of the mitochondrial membrane uncoupler FCCP. As we are using cell number for normalization, the apparent increases in oxidative phosphorylation and maximal oxygen consumption may reflect the demonstrated increase of mitochondria in MRL muscle cells. However, the data remain pertinent as it demonstrates that the MRL cells have increased oxidative phosphorylation and an increase in physiologic reserve. As glycolysis – indicated by acidification – occurs in the cytoplasm this increase likely reflects a true cellular rate change. Similarly, the MRL muscles have slightly decreased ratios of oxygen consumption to acidification rate. This further indicates that the MRL muscle cells have a higher ratio of glycolysis to oxidative phosphorylation than the B6 control muscle cells do.

## Conclusion

We undertook these experiments to identify molecular metabolic differences between the fibrosis‐resistant, super healing MRL mice, and the standard control B6 mice. We have found that the MRL mice have increased mitochondria and increased pAMPK to AMPK ratio. The data presented here allow us to put forth a model of altered metabolism in the skeletal muscle of the MRL mouse. We hypothesize that these mice are inefficient at producing ATP by the normal methods – substrate level phosphorylation of the Kreb's cycle and oxidative phosphorylation by the electron transport chain – and, therefore, must compensate. We argue that the compensation has multiple avenues. The MRL mice perform more glycolysis (Naviaux et al. 2009), which is usually associated with short‐term compensation as is required in acute exercise. The mice also have increased mitochondrial content and increased pAMPK which may be viewed as chronic adaptations to low energy levels. Although as pAMPK remains elevated the mice never fully compensate for their energy insufficiency. The mice also retain some embryonic markers and have decreased ROS (Naviaux et al. 2009). These many unique characteristics possibly increase the healing capabilities of these mice, although separating the salient differences from the bystander differences continues to prove challenging.

An abundance of studies with increased pAMPK either through exercise, transgenics or pharmacueticals have recently been published. With this novel MRL mouse strain data, the scientific community now also has a mouse strain with endogenously and chronically increased activated AMPK. AMPK activation is increasingly becoming discussed as an attractive therapeutic target to halt muscle wasting diseases. Roles for AMPK have been identified in mouse muscle cell autophagy and maintenance (reviewed in (Sanchez et al. 2012)), increasing utrophin expression for treatment of dystrophin mutant muscular dystrophy (Ljubicic et al. 2011), and halting muscular dystrophy pathology (Ljubicic et al. 2011; Bueno Junior et al. 2012; Jahnke et al. 2012; Pauly et al. 2012). Furthermore, increased mitochondrial content – as shown in the MRL muscles – may be of additional benefit in Muscular Dystrophy due to their ability to buffer the inappropriate calcium signaling occurring in MD muscles (Selsby et al. 2012). It is also becoming increasingly obvious that therapeutics utilized to combat type 2 diabetes require pAMPK for function (Viollet et al. 2012). As type 2 diabetes is ultimately a disease of skeletal muscle this syndrome can also be considered in our list of muscle wasting diseases that respond favorably to AMPK activators.

The naturally increased pAMPK in MRL skeletal muscle tissue is of particular interest for the muscular dystrophy field. We have recently demonstrated that F2 MRL by DBA2/J intercross mice with a muscular dystrophy causing *γ*–sarcoglycan null mutation (Sgcg−/−) have a very mild disease with significantly reduced fibrosis in skeletal, diaphragm, and cardiac muscle compared to DBA2/J Sgcg−/− parental strain (Heydemann et al. 2012). Furthermore, the F2 Sgcg−/− mice, with only 50% of MRL contributed nuclear genome, have significantly better cardiac function. Now, in this current manuscript, we have identified a candidate molecular mechanism behind the MRL's mild muscular dystrophy disease. That increasing pAMPK will aid muscular dystrophy mouse models are becoming accepted. However, how increased pAMPK benefits the mice is still under scrutiny. We will continue to investigate the unique MRL mice and their unique metabolism to further the understanding of how increasing pAMPK is beneficial. Identifying the specific pAMPK targets which diminish muscular dystrophy is important for the identification of more specific therapies. In addition, therapies for other muscle diseases may become revealed with these mechanistic investigations.

## Conflict of Interest

The authors have no conflicts, financial or otherwise to declare.

## References

[phy2252-bib-0001] Adachi, M. , R. Watanabe‐Fukunaga , and S. Nagata . 1993 Aberrant transcription caused by the insertion of an early transposable element in an intron of the Fas antigen gene of lpr mice. Proc. Natl Acad. Sci. USA 90:1756–1760. 7680478 10.1073/pnas.90.5.1756 PMC45958

[phy2252-bib-0002] Alleva, D. G. , S. B. Kaser , and D. I. Beller . 1997 Aberrant cytokine expression and autocrine regulation characterize macrophages from young MRL+/+ and NZB/W F1 lupus‐prone mice. J. Immunol. 159:5610–5619. 9548504

[phy2252-bib-0003] Arthur, L. M. , R. M. Demarest , L. Clark , D. Gourevitch , K. Bedelbaeva , R. Anderson , et al. 2010 Epimorphic regeneration in mice is p53‐independent. Cell Cycle 9:3667–3673. 20855943 10.4161/cc.9.18.13119 PMC3047795

[phy2252-bib-0004] Baker, K. L. , S. B. Daniels , J. B. Lennington , T. Lardaro , A. Czap , R. Q. Notti , et al. 2006 Neuroblast protuberances in the subventricular zone of the regenerative MRL/MpJ mouse. J. Comp. Neurol. 498:747–761. 16927265 10.1002/cne.21090

[phy2252-bib-0005] Bedelbaeva, K. , A. Snyder , D. Gourevitch , L. Clark , X. M. Zhang , J. Leferovich , et al. 2010 Lack of p21 expression links cell cycle control and appendage regeneration in mice. Proc. Natl Acad. Sci. USA 107:5845–5850. 20231440 10.1073/pnas.1000830107 PMC2851923

[phy2252-bib-0006] Bueno Junior, C. R. , L. C. Pantaleao , V. A. Voltarelli , L. H. Bozi , P. C. Brum , and M. Zatz . 2012 Combined effect of AMPK/PPAR agonists and exercise training in mdx mice functional performance. PLoS ONE 7:e45699. 23029189 10.1371/journal.pone.0045699 PMC3448675

[phy2252-bib-0007] Clark, L. D. , R. K. Clark , and E. Heber‐Katz . 1998 A new murine model for mammalian wound repair and regeneration. Clin. Immunol. Immunopathol. 88:35–45. 9683548 10.1006/clin.1998.4519

[phy2252-bib-0008] Corton, J. M. , J. G. Gillespie , S. A. Hawley , and D. G. Hardie . 1995 5‐aminoimidazole‐4‐carboxamide ribonucleoside. A specific method for activating AMP‐activated protein kinase in intact cells? Eur. J. Biochem. 229:558–565. 7744080 10.1111/j.1432-1033.1995.tb20498.x

[phy2252-bib-0009] Davies, S. P. , N. R. Helps , P. T. Cohen , and D. G. Hardie . 1995 5′‐AMP inhibits dephosphorylation, as well as promoting phosphorylation, of the AMP‐activated protein kinase. Studies using bacterially expressed human protein phosphatase‐2C alpha and native bovine protein phosphatase‐2AC. FEBS Lett. 377:421–425. 8549768 10.1016/0014-5793(95)01368-7

[phy2252-bib-0010] Donnelly, R. P. , J. Levine , D. Q. Hartwell , G. Frendl , M. J. Fenton , and D. I. Beller . 1990 Aberrant regulation of IL‐1 expression in macrophages from young autoimmune‐prone mice. J. Immunol. 145:3231–3239. 2230116

[phy2252-bib-0011] Droge, W. 2002 Free radicals in the physiological control of cell function. Physiol. Rev. 82:47–95. 11773609 10.1152/physrev.00018.2001

[phy2252-bib-0012] Foretz, M. , S. Hebrard , J. Leclerc , E. Zarrinpashneh , M. Soty , G. Mithieux , et al. 2010 Metformin inhibits hepatic gluconeogenesis in mice independently of the LKB1/AMPK pathway via a decrease in hepatic energy state. J. Clin. Invest. 120:2355–2369. 20577053 10.1172/JCI40671 PMC2898585

[phy2252-bib-0013] Garcia‐Cazarin, M. L. , J. L. Gamboa , and F. H. Andrade . 2011 Rat diaphragm mitochondria have lower intrinsic respiratory rates than mitochondria in limb muscles. Am. J. Physiol. Regul. Integr. Comp. Physiol. 300:R1311–R1315. 21389333 10.1152/ajpregu.00203.2010 PMC3119153

[phy2252-bib-0014] Ghezzi, D. , E. Baruffini , T. B. Haack , F. Invernizzi , L. Melchionda , C. Dallabona , et al. 2012 Mutations of the mitochondrial‐tRNA modifier MTO1 cause hypertrophic cardiomyopathy and lactic acidosis. Am. J. Hum. Genet. 90:1079–1087. 22608499 10.1016/j.ajhg.2012.04.011 PMC3370278

[phy2252-bib-0015] Gilliam, L. A. , K. H. Fisher‐Wellman , C. T. Lin , J. M. Maples , B. L. Cathey , and P. D. Neufer . 2013 The anticancer agent doxorubicin disrupts mitochondrial energy metabolism and redox balance in skeletal muscle. Free Radic. Biol. Med. 65:988–996. 24017970 10.1016/j.freeradbiomed.2013.08.191 PMC3859698

[phy2252-bib-0016] Godin, R. , F. Daussin , S. Matecki , T. Li , B. J. Petrof , and Y. Burelle . 2012 Peroxisome proliferator‐activated receptor gamma coactivator1‐ gene alpha transfer restores mitochondrial biomass and improves mitochondrial calcium handling in post‐necrotic mdx mouse skeletal muscle. J. Physiol. 590(Pt 21):5487–5502. 22907054 10.1113/jphysiol.2012.240390 PMC3515833

[phy2252-bib-0017] Hardie, D. G. 2003 Minireview: the AMP‐activated protein kinase cascade: the key sensor of cellular energy status. Endocrinology 144:5179–5183. 12960015 10.1210/en.2003-0982

[phy2252-bib-0018] Hardie, D. G. 2011 Sensing of energy and nutrients by AMP‐activated protein kinase. Am. J. Clin. Nutr. 93:891S–896S. 21325438 10.3945/ajcn.110.001925

[phy2252-bib-0019] Heber‐Katz, E. , J. Leferovich , K. Bedelbaeva , D. Gourevitch , and L. Clark . 2004 The scarless heart and the MRL mouse. Philos. Trans. R. Soc. Lond. B Biol. Sci. 359:785–793. 15293806 10.1098/rstb.2004.1468 PMC1693365

[phy2252-bib-0020] Heydemann, A. 2012 The super super‐healing MRL mouse strain. Front. Biol. 7:522–538. 10.1007/s11515-012-1192-4 PMC3806350 24163690

[phy2252-bib-0021] Heydemann, A. , K. A. Swaggart , G. H. Kim , J. Holley‐Cuthrell , M. Hadhazy , and E. M. McNally . 2012 The superhealing MRL background improves muscular dystrophy. Skelet. Muscle 2:26. 23216833 10.1186/2044-5040-2-26 PMC3534636

[phy2252-bib-0022] Hiona, A. , A. Sanz , C. Kujoth , R. Pamplona , A. Y. Seo , T. Hofer , et al. 2010 Mitochondrial DNA mutations induce mitochondrial dysfunction, apoptosis and sarcopenia in skeletal muscle of mitochondrial DNA mutator mice. PLoS ONE 5:e11468. 20628647 10.1371/journal.pone.0011468 PMC2898813

[phy2252-bib-0023] Irimia, J. M. , J. Rovira , J. N. Nielsen , M. Guerrero , J. F. Wojtaszewski , and R. Cusso . 2012 Hexokinase 2, glycogen synthase and phosphorylase play a key role in muscle glycogen supercompensation. PLoS ONE 7:e42453. 22860128 10.1371/journal.pone.0042453 PMC3409157

[phy2252-bib-0024] Irwin, M. H. , K. Parameshwaran , and C. A. Pinkert . 2012 Mouse models of mitochondrial complex I dysfunction. Int. J. Biochem. Cell Biol. 45:34–40. 22903069 10.1016/j.biocel.2012.08.009 PMC3508304

[phy2252-bib-0025] Jahnke, V. E. , J. H. Van Der Meulen , H. K. Johnston , S. Ghimbovschi , T. Partridge , E. P. Hoffman , et al. 2012 Metabolic remodeling agents show beneficial effects in the dystrophin‐deficient mdx mouse model. Skelet. Muscle 2:16. 22908954 10.1186/2044-5040-2-16 PMC3482394

[phy2252-bib-0026] Juvet, S. C. , C. W. Thomson , E. Y. Kim , B. Joe , O. Adeyi , and L. Zhang . 2013 FcRgamma promotes T cell apoptosis in Fas‐deficient mice. J. Autoimmun. 42:80–93. 23313147 10.1016/j.jaut.2012.12.002

[phy2252-bib-0027] Kanki, T. , K. Ohgaki , M. Gaspari , C. M. Gustafsson , A. Fukuoh , N. Sasaki , et al. 2004 Architectural role of mitochondrial transcription factor A in maintenance of human mitochondrial DNA. Mol. Cell. Biol. 24:9823–9834. 15509786 10.1128/MCB.24.22.9823-9834.2004 PMC525493

[phy2252-bib-0028] Kench, J. A. , D. M. Russell , V. A. Fadok , S. K. Young , G. S. Worthen , J. Jones‐Carson , et al. 1999 Aberrant wound healing and TGF‐beta production in the autoimmune‐prone MRL/+ mouse. Clin. Immunol. 92:300–310. 10479535 10.1006/clim.1999.4754

[phy2252-bib-0029] Kong, X. , R. Wang , Y. Xue , X. Liu , H. Zhang , Y. Chen , et al. 2010 Sirtuin 3, a new target of PGC‐1alpha, plays an important role in the suppression of ROS and mitochondrial biogenesis. PLoS ONE 5:e11707. 20661474 10.1371/journal.pone.0011707 PMC2908542

[phy2252-bib-0030] Kudo, N. , A. J. Barr , R. L. Barr , S. Desai , and G. D. Lopaschuk . 1995 High rates of fatty acid oxidation during reperfusion of ischemic hearts are associated with a decrease in malonyl‐CoA levels due to an increase in 5′‐AMP‐activated protein kinase inhibition of acetyl‐CoA carboxylase. J. Biol. Chem. 270:17513–17520. 7615556 10.1074/jbc.270.29.17513

[phy2252-bib-0031] Lagouge, M. , C. Argmann , Z. Gerhart‐Hines , H. Meziane , C. Lerin , F. Daussin , et al. 2006 Resveratrol improves mitochondrial function and protects against metabolic disease by activating SIRT1 and PGC‐1alpha. Cell 127:1109–1122. 17112576 10.1016/j.cell.2006.11.013

[phy2252-bib-0032] Ljubicic, V. , P. Miura , M. Burt , L. Boudreault , S. Khogali , J. A. Lunde , et al. 2011 Chronic AMPK activation evokes the slow, oxidative myogenic program and triggers beneficial adaptations in mdx mouse skeletal muscle. Hum. Mol. Genet. 20:3478–3493. 21659335 10.1093/hmg/ddr265

[phy2252-bib-0033] Lombardi, A. , R. De Matteis , M. Moreno , L. Napolitano , R. A. Busiello , R. Senese , et al. 2012 Responses of skeletal muscle lipid metabolism in rat gastrocnemius to hypothyroidism and iodothyronine administration: a putative role for FAT/CD36. Am. J. Physiol. Endocrinol. Metab. 303:E1222–E1233. 22967501 10.1152/ajpendo.00037.2012

[phy2252-bib-0034] Moreno‐Loshuertos, R. , R. Acin‐Perez , P. Fernandez‐Silva , N. Movilla , A. Perez‐Martos , S. Rodriguez de Cordoba , et al. 2006 Differences in reactive oxygen species production explain the phenotypes associated with common mouse mitochondrial DNA variants. Nat. Genet. 38:1261–1268. 17013393 10.1038/ng1897

[phy2252-bib-0035] Naviaux, R. K. , T. P. Le , K. Bedelbaeva , J. Leferovich , D. Gourevitch , P. Sachadyn , et al. 2009 Retained features of embryonic metabolism in the adult MRL mouse. Mol. Genet. Metab. 96:133–144. 19131261 10.1016/j.ymgme.2008.11.164 PMC3646557

[phy2252-bib-0036] Pauly, M. , F. Daussin , Y. Burelle , T. Li , R. Godin , J. Fauconnier , et al. 2012 AMPK activation stimulates autophagy and ameliorates muscular dystrophy in the mdx mouse diaphragm. Am. J. Pathol. 181:583–592. 22683340 10.1016/j.ajpath.2012.04.004

[phy2252-bib-0037] Ponticos, M. , Q. L. Lu , J. E. Morgan , D. G. Hardie , T. A. Partridge , and D. Carling . 1998 Dual regulation of the AMP‐activated protein kinase provides a novel mechanism for the control of creatine kinase in skeletal muscle. EMBO J. 17:1688–1699. 9501090 10.1093/emboj/17.6.1688 PMC1170516

[phy2252-bib-0038] Qiu, Q. , R. Li , P. Jiang , L. Xue , Y. Lu , Y. Song , et al. 2012 Mitochondrial tRNA mutations are associated with maternally inherited hypertension in two Han Chinese pedigrees. Hum. Mutat. 33:1285–1293. 22549939 10.1002/humu.22109

[phy2252-bib-0039] Rando, T. A. , and H. M. Blau . 1994 Primary mouse myoblast purification, characterization, and transplantation for cell‐mediated gene therapy. J. Cell Biol. 125:1275–1287. 8207057 10.1083/jcb.125.6.1275 PMC2290930

[phy2252-bib-0040] Sachadyn, P. , X. M. Zhang , L. D. Clark , R. K. Naviaux , and E. Heber‐Katz . 2008 Naturally occurring mitochondrial DNA heteroplasmy in the MRL mouse. Mitochondrion 8:358–366. 18761428 10.1016/j.mito.2008.07.007 PMC2631412

[phy2252-bib-0041] Salt, I. P. , G. Johnson , S. J. Ashcroft , and D. G. Hardie . 1998 AMP‐activated protein kinase is activated by low glucose in cell lines derived from pancreatic beta cells, and may regulate insulin release. Biochem. J. 335(Pt 3):533–539. 9794792 10.1042/bj3350533 PMC1219813

[phy2252-bib-0042] Sanchez, A. M. , A. Csibi , A. Raibon , K. Cornille , S. Gay , H. Bernardi , et al. 2012 AMPK promotes skeletal muscle autophagy through activation of forkhead FoxO3a and interaction with Ulk1. J. Cell. Biochem. 113:695–710. 22006269 10.1002/jcb.23399

[phy2252-bib-0043] Selsby, J. T. , K. J. Morine , K. Pendrak , E. R. Barton , and H. L. Sweeney . 2012 Rescue of dystrophic skeletal muscle by PGC‐1alpha involves a fast to slow fiber type shift in the mdx mouse. PLoS ONE 7:e30063. 22253880 10.1371/journal.pone.0030063 PMC3256197

[phy2252-bib-0044] Szczepanowska, J. , D. Malinska , M. R. Wieckowski , and J. Duszynski . 2012 Effect of mtDNA point mutations on cellular bioenergetics. Biochim. Biophys. Acta 1817:1740–1746. 22406627 10.1016/j.bbabio.2012.02.028

[phy2252-bib-0045] Taub, P. R. , I. Ramirez‐Sanchez , T. P. Ciaraldi , G. Perkins , A. N. Murphy , R. Naviaux , et al. 2012 Alterations in skeletal muscle indicators of mitochondrial structure and biogenesis in patients with type 2 diabetes and heart failure: effects of epicatechin rich cocoa. Clin. Transl. Sci. 5:43–47. 22376256 10.1111/j.1752-8062.2011.00357.x PMC5439909

[phy2252-bib-0046] Theofilopoulos, A. N. , and F. J. Dixon . 1985 Murine models of systemic lupus erythematosus. Adv. Immunol. 37:269–390. 3890479 10.1016/s0065-2776(08)60342-9

[phy2252-bib-0047] Trifunovic, A. , A. Hansson , A. Wredenberg , A. T. Rovio , E. Dufour , I. Khvorostov , et al. 2005 Somatic mtDNA mutations cause aging phenotypes without affecting reactive oxygen species production. Proc. Natl. Acad. Sci. U.S.A. 102:17993–17998. 16332961 10.1073/pnas.0508886102 PMC1312403

[phy2252-bib-0048] Ueno, M. , B. L. Lyons , L. M. Burzenski , B. Gott , D. J. Shaffer , D. C. Roopenian , et al. 2005 Accelerated wound healing of alkali‐burned corneas in MRL mice is associated with a reduced inflammatory signature. Invest. Ophthalmol. Vis. Sci. 46:4097–4106. 16249486 10.1167/iovs.05-0548

[phy2252-bib-0049] Viollet, B. , B. Guigas , N. Sanz Garcia , J. Leclerc , M. Foretz , and F. Andreelli . 2012 Cellular and molecular mechanisms of metformin: an overview. Clin. Sci. (Lond) 122:253–270. 22117616 10.1042/CS20110386 PMC3398862

[phy2252-bib-0050] Virbasius, J. V. , and R. C. Scarpulla . 1994 Activation of the human mitochondrial transcription factor A gene by nuclear respiratory factors: a potential regulatory link between nuclear and mitochondrial gene expression in organelle biogenesis. Proc. Natl. Acad. Sci. U.S.A. 91:1309–1313. 8108407 10.1073/pnas.91.4.1309 PMC43147

[phy2252-bib-0051] Winder, W. W. , and D. G. Hardie . 1996 Inactivation of acetyl‐CoA carboxylase and activation of AMP‐activated protein kinase in muscle during exercise. Am. J. Physiol. 270(2 Pt 1):E299–E304. 8779952 10.1152/ajpendo.1996.270.2.E299

[phy2252-bib-0052] Zong, H. , J. M. Ren , L. H. Young , M. Pypaert , J. Mu , M. J. Birnbaum , et al. 2002 AMP kinase is required for mitochondrial biogenesis in skeletal muscle in response to chronic energy deprivation. Proc. Natl. Acad. Sci. U.S.A. 99:15983–15987. 12444247 10.1073/pnas.252625599 PMC138551

